# Evaluation of the correlation between depression and physical activity among older persons with osteoporosis: a cross-sectional study

**DOI:** 10.3389/fpsyt.2023.1193072

**Published:** 2023-08-30

**Authors:** Linjun Shi, Xiaoping Zhou, Yueshan Gao, Xia Li, Ronghua Fang, Xuexue Deng

**Affiliations:** ^1^West China School of Nursing/General Practice Ward, International Medical Center Ward, General Practice Medical Center, West China Hospital, Sichuan University, Chengdu, China; ^2^West China School of Nursing, Sichuan University, Chengdu, China; ^3^West China School of Nursing/Department of Nursing, West China Hospital, Sichuan University, Chengdu, China

**Keywords:** osteoporosis, older persons, depression, physical activity, cross-sectional study

## Abstract

**Background:**

Osteoporosis is a common chronic disease in older persons. Physical activity can prevent chronic diseases as well as many diseases associated with aging. Older persons often suffer from depression and other psychological problems. However, there are few studies on the correlation between depression and physical activity in older persons with osteoporosis in China.

**Methods:**

This cross-sectional study was conducted from June 1 to December 28, 2021. A total of 207 subjects who met the inclusion and were exclusion criteria were selected from the outpatient department of West China Hospital and evaluated using a self-designed demographic data questionnaire, the Self-rating Depression Scale (SDS), and the Physical Activity Scale for the Elderly (PASE). Multivariate linear regression was performed to analyze the factors affecting physical activity, and multivariate logistic regression analysis was performed to analyze the factors affecting depression. Spearman’s correlation coefficients were calculated to analyze the correlation between depression and physical activity in older persons with osteoporosis.

**Results:**

A total of 173 valid questionnaires were statistically analyzed. A total of 122 (70.5%) participants were identified as having depression (SDS ≥ 50 points). The median depression score was 62.5 (24.38), and the median PASE score was 69.29 (116.64). Multivariate logistic regression model results showed that physical activity and pain were the main risk factors for depression in older persons with osteoporosis (*p* < 0.05). Multivariate linear regression model results showed that gender, participation in social activities, activities of daily living status (ADLs), and depression status were the main risk factors associated with physical activity in older persons with osteoporosis (*p* < 0.05). The results of the correlation analysis showed that physical activity was negatively correlated with depression (*R* = −0.510, *p* = 0.000).

**Conclusion:**

We found that depression was negatively correlated with physical activity in older persons with osteoporosis in China. This means that the less physical activity there is, the more serious the depression status and having depression may result in reduced physical activity in older persons with osteoporosis. To better help older persons with osteoporosis, medical staff should give more attention to influencing factors of depression and physical activity.

## 1. Background

Osteoporosis (OP) is an age-related disease. The prevalence of OP has been reported to be 36% in the general Chinese population aged 60 years or older 49% in women, and 23% in men (1). OP is associated with an increased risk for fractures, which can lead to disability, pain, loss of functional independence, and increases in morbidity, socioeconomic stress, and mortality (2, 3). The number of persons with osteoporotic fractures is expected to increase from 2.69 million in 2015 to 5.99 million in 2050, and the corresponding medical expenditures are predicted to be as high as 254 million dollars in China (4, 5). Stereoscopic fracture is the major complication of OP, and without medical intervention, the lifetime risk is within the range of 40–50% in females and 13–22% in males (6).

Physical activity (PA) can prevent fractures by improving muscle mass and balance and by increasing skeletal strength, thus decreasing the risk of injurious falls (7, 8) and reducing the risk of fractures (9). Epidemiological data have indicated that depression affects the older population at a rate of 24.9% in China (10) and 10–20% worldwide (11). Depression mainly affects those with chronic diseases and cognitive impairment; it causes suffering, family breakdown, and disability, exacerbates the consequences of many diseases, and it increases mortality in elderly people (12). Depression is a leading cause of disability and a burden on health care systems worldwide (13). A previous study reported that 33.0% of female OP patients in Germany were depressed (14), and in another study, 69.1% of elderly people with OP were reported to be depressed (15). A previous study showed that 56% of persons with OP in China, had negative emotions, such as anxiety and depression (16), and the prevalence of depression in elderly adults with OP was higher than that in elderly persons without OP (10).

The literature has demonstrated that depression may be associated with reduced physical activity (17, 18). Conversely, PA can bring about changes in endorphin and monoamine levels or a decrease in the level of cortisol, a stress hormone, which may result in an improvement in a person’s mood (19). Therefore, PA is a protective factor against depression, and active older adults have a better quality of life and fewer symptoms of depression than their sedentary peers (20). Research by Chinese scholars showed that older persons with OP experienced aggravated psychological fear and avoidance behavior due to chronic pain and fear of exercise caused by an injury (21).

Older persons with depression may reduce their PA, and low PA is associated with decreased BMD, thus increasing the risk of fractures (22). To date, an increasing number of studies have been conducted on depression and PA in older adults with OP. However, there are few reports on the correlation between depression and PA in older persons with OP in China at present.

Therefore, this study aims to evaluate the correlation between depression and PA among older persons with OP.

## 2. Methods

### 2.1. Study population

All study participants underwent for bone mineral density (BMD) measurement by dual-energy X-ray absorptiometry (DEXA), and OP diagnoses were consistent with the Chinese guidelines for the diagnosis and treatment of senile OP (2018 edition) (23). OP is a systemic skeletal disease characterized by low bone mass and microarchitectural deterioration of bone tissue bone mineral density (BMD) measured by dual-energy X-ray absorptiometry (DEXA). DEXA scans are the most commonly used diagnostic method for OP, and the findings are usually expressed by a T Score: T ≥ −1.0 is normal; −2.5 < T < −1.0 is low bone; T ≤ −2.5 is OP; and severe OP is T ≤ −2.5 with fragility fracture (23). The inclusion criteria were as follows: age ≥ 60 years, diagnosis of OP, and informed and voluntary willingness to participate in the survey. The exclusion criteria were as follows: cognitive impairment; inability to understand questionnaire content; comorbidities that affect activity, such as malignant disease, thyroid, parathyroid or adrenal gland disease; and a history of depression.

### 2.2. Data collection

Convenience sampling was used in this study. A cross-sectional study using questionnaires was performed among older persons with OP in the outpatient department of West China Hospital from Between June 1, to December 28, 2021. For data collection, the number of participants should be 10 times the number of independent variables in studies that investigate a correlation between 2 variables or multivariate variables (24). In this study, a multivariate analysis was chosen to assess the relationship of 17 independent variables. Therefore, the minimum required sample size was at least 170. Each participant completed three questionnaires: a questionnaire assessing demographic data, the Self-rating Depression Scale (SDS), and the Physical Activity Scale for the Elderly (PASE). Members of the research team interviewed each participant in the outpatient department of West China Hospital of Sichuan University. The trained investigators used uniform guidelines to explain the purpose of the study and its significance as well as the questionnaire completion methods to the participants; then, participants gave their consent and signed the informed consent form before the survey. Participants completed the questionnaires independently, and the questionnaires were collected by the researchers immediately after completion.

The data were verified and entered by two researchers, and a database was built using EpiData 3.1 software (EpiData–Comprehensive Data Management and Basic Statistical Analysis System, EpiData Association, Odense, Denmark), Then, the data were double-checked. Incomplete questionnaires with missing items were deleted.

### 2.3. Measures

Depression was assessed using Zung’s SDS (25). The Chinese version of Cronbach’s α was 0.784 (26). The SDS is composed of 20 items and assesses the frequency of symptoms in the past week using a 4-point Likert scale: none of the time or rarely, some of the time, a good part of the time, most or all of the time. In the 20 items, there are 10 positive and 10 negative items, which are assigned a score of 1, 2, 3, or 4 for the positive items and 4, 3, 2, or 1 for the negative items. The 20 item scores are added to obtain the original scores, which are multiplied by 1.25 to calculate the standard score. Higher scores indicate worse depression. In this study, depression scores were classified as follows: without depression, < 50 points, and with depression, ≥ 50 points (27).

The PASE was used to assess the PA of the participants. The PASE was developed by Washburn et al. (28). The test–retest reliability, which was assessed over a 3–7 week interval, was 0.75 (28). The questionnaire includes 12 items that are used to assess PA over the past 7 days based on 3 domains of activity: leisure, household, and work-related activity. Leisure PA includes the following activities: walking outside: light, moderate, and strenuous sports; and activities aimed at muscle strength or endurance exercise. The possible responses for each question are “never,” “seldom,” “sometimes,” or “often.” Respondents were asked to indicate the number of days per week they engaged in each activity and then the number of hours per day. The type of activity performed and the average time spent on that activity each day were recorded. Household PA includes light housework, heavy housework, home repairs, lawn work or yard care, outdoor gardening, and taking care of others, and those items use a “yes” or “no” response format. Work-related PA includes work for pay or as a volunteer and the hours of work involved. PASE scores are from weights and frequency values for each of 12 types of activity. The calculation is performed by multiplying the amount of time spent (hours/day over a seven-day period) in each participation category by the PASE weighted value and then summing each contribution for a total score. The total score ranges from 0 to 400 or more. The higher the score is, the higher the level of PA (29).

### 2.4. Covariates

According to the results of the survey, the demographic data were classified into the following categories: age group (60–74, ≥ 75); gender (male, female); marital status (married, divorced, or widowed); education (primary school and below, junior high school, high school, college and above); past occupation (manual labor, intellectual labor); cohabitation status (alone or nursing home, cohabitation with spouse, cohabitation with child); and income (pension or social security, child support, others).

Health situation factors were measured as follows. BMI was obtained as the weight (kg) divided by the square of the height (m). We categorized BMI into four groups: underweight, BMI < 18.5 kg/m^2^; normal weight, BMI 18.5–23.9 kg/m^2^; overweight, BMI 24–27.9 kg/m^2^; and obesity, BMI ≥ 28 kg/m^2^ (30). Other factors included smoking history (no or yes); alcohol consumption history (no or yes); pain (no or yes); and history of fracture (no or yes). Comorbidity was defined as the presence of ≥2 conditions (no or yes). Polypharmacy was defined as simultaneously taking ≥5 medications (no or yes). Participation in social activities was categorized as none, 1–2 times/year, 1–2 times/month, or 1–2 times/week. The modified Barthel index (MBI) was used to assess activities of daily living (ADLs). ADLs includes feeding, dressing, grooming, bathing, bowel and bladder control, toilet use, transfer, mobility, and climbing stairs. The total score ranges from 0 to 100, with lower scores indicating worse ADL impairment. We divided ADL into four statuses: no impairment = 100; mild impairment = 61–99; moderate impairment = 41–60; and severe impairment = 0–40 (31, 32). The frailty phenotype scale by Fried et al. (33), was used to assess frailty, and it includes five domains: weight loss, self-reported exhaustion, slow walking speed, weakness, and low PA. Each domain is assigned 0–1 points, with a range of 0–5 points: non-frail = 1, prefrail = 2, and frail = 3 (33).

### 2.5. Statistical analysis

SPSS software (Version 20.0. IBM Inc., Armonk, NY) was used for statistical analyses. Categorical data are presented as frequencies and percentages. Continuous data conforming to a normal distribution are presented as the means and standard deviations, and *T*-tests or ANOVA were used for comparisons between groups. Continuous data that did not conform to a normal distribution are represented by median and interquartile range, and nonparametric tests were used for comparisons between groups. Multivariate logistic regression was used to analyze the influencing factors of depression. Depression status (i.e., with or without depression) was the dependent variable, and the independent variables included those that were significant (*p* < 0.2) in the univariate analysis, including demographic data (age, gender, marital status, education, past occupation, cohabitation status, income, BMI, smoking history, alcohol consumption history, pain, history of fracture, comorbidity, polypharmacy, participation in social activities, ADLs, and frailty status) and PA scores. Multivariate linear regression was used to analyze the influencing factors of PA. PA scores were the dependent variable, and considering the interference factors, the independent variables included the demographic variables that were significant (*p* < 0.2) in the univariate analysis (age, sex, marital status, education, past occupation, cohabitation status, income, BMI, smoking history, alcohol consumption history, pain, history of fracture, comorbidity, polypharmacy, participation in social activities, ADLs, frailty status and depression). The correlation between depression and PA was analyzed. Pearson correlation analysis was used if the data followed a normal distribution, and Spearman correlation analysis was used if the data did not follow a normal distribution. A *p* < 0.05 was considered to indicate statistical significance.

## 3. Results

### 3.1. Participant characteristics

207 subjects completed the questionnaire, and the number of effective questionnaires (questionnaires included in the analysis) was 173. Effective questionnaire recovery rate was 83.57%. The participants ranged in age from 60 to 94 years, with an average age of 70.63 ± 9.02 years. The sample also had the following characteristics: 60–74 years old, 65.9%; female, 83.2%; married, 79.8%; in pain, 91.3%; comorbidity, 70.5%; no participation in social activities, 45.1%; frail, 71.7%; and depressed, 70.5% (Table 1).

**Table 1:** Comparison of depression and PA scores with different demographic characteristics of participants (*n* = 173, in Sichuan, China, 2021).

Variables	n (%)	Depression scores median (IQR)	Z/H	*P*	PA scores median (IQR)	Z/H	*P*
Age, y			−2.768	0.006		−6.393	0.000
60–74	114 (65.9)	61.25 (22.81)			102.50 (110.00)		
≥75	59 (34.1)	67.50 (20.00)			4.00 (62.00)		
Gender			−0.756	0.450		−0.645	0.519
Male	29 (16.8)	57.50 (25.63)			34.00 (131.50)		
Female	144 (83.2)	63.75 (23.75)			74.00 (124.50)		
Marital status			−1.183	0.237		−2.931	0.003
Married	138 (79.8)	62.50 (21.25)			80.00 (121.00)		
Divorced/Widowed	35 (20.2)	68.75 (28.75)			10.00 (83.00)		
Education			5.506	0.138		10.260	0.016
Primary school and below	61 (35.3)	63.75 (21.88)			42.00 (111.00)		
Junior high school	48 (27.7)	63.75 (27.50)			45.00 (96.75)		
High school	39 (22.5)	63.75 (18.75)			95.00 (118.00)		
College and above	25 (14.5)	55.00 (24.38)			124.00 (142.50)		
Past occupation			−0.775	0.438		−0.145	0.884
Manual labor	110 (63.6)	61.25 (27.81)			72.00 (137.00)		
Intellectual labor	63 (36.4)	65.00 (17.50)			65.00 (116.00)		
Cohabitation status			17.924	0.000		16.467	0.000
Alone/Nursing home	13 (7.5)	63.75 (38.75)			87.00 (171.00)		
Cohabitation with spouse	65 (37.6)	58.75 (20.00)			95.00 (97.00)		
Cohabitation with child	95 (54.9)	67.50 (23.75)			34.00 (102.00)		
Income			12.814	0.002		6.232	0.044
Pension/Social security	106 (61.3)	62.50 (23.75)			74.00 (131.25)		
Child support	57 (32.9)	66.25 (22.50)			35.00 (107.50)		
Others	10 (5.8)	46.88 (15.63)			120.50 (55.25)		
BMI (kg/m^2^)			2.747	0.432		0.643	0.886
Underweight	18 (10.4)	56.88 (25.00)			92.50 (136.50)		
Normal weight	95 (54.9)	62.50 (23.75)			65.00 (131.00)		
Overweight	51 (29.5)	63.75 (25.00)			69.00 (117.00)		
Obesity	9 (5.2)	66.25 (20.00)			62.00 (122.50)		
Smoking history			−0.013	0.990		−1.793	0.073
No	157 (90.8)	62.50 (23.13)			70.00 (130.50)		
Yes	16 (9.2)	65.00 (27.19)			17.50 (98.50)		
Alcohol consumption history			−0.191	0.848		−0.851	0.395
No	157 (90.8)	62.50 (23.75)			70.00 (121.50)		
Yes	16 (9.2)	65.00 (25.63)			19.00 (147.75)		
Pain			−4.593	0.000		−3.651	0.000
No	15 (8.7)	36.25 (16.25)			150.00 (70.00)		
Yes	158 (91.3)	63.75 (20.63)			55.00 (116.00)		
History of fracture			−0.987	0.324		−1.394	0.163
No	112 (64.7)	62.50 (22.50)			79.00 (131.00)		
Yes	61 (35.3)	63.75 (23.13)			54.00 (118.50)		
Comorbidity			−2.543	0.011		−1.086	0.278
No	51 (29.5)	60.00 (21.25)			79.00 (140.00)		
Yes	122 (70.5)	64.38 (26.25)			66.00 (116.00)		
Polypharmacy			−5.754	0.000		−3.983	0.000
No	139 (80.3)	58.75 (21.25)			81.00 (127.00)		
Yes	34 (19.7)	75.00 (13.75)			15.00 (55.50)		
Participation in social activities			14.920	0.002		27.400	0.000
No	78 (45.1)	68.75 (27.81)			37.00 (93.75)		
1–2 times/year	76 (43.9)	61.25 (18.75)			80.00 (138.75)		
1–2 times/month	12 (6.9)	56.25 (26.56)			141.50 (109.50)		
1–2 times/week	7 (4.0)	50.00 (33.75)			175.00 (45.00)		
ADLs			33.912	0.000		76.173	0.000
No impairment	57 (32.9)	51.25 (26.88)			126.00 (73.00)		
Mild impairment	89 (51.4)	63.75 (18.75)			49.00 (91.00)		
Moderate impairment	18 (10.4)	71.25 (10.31)			0.00 (4.00)		
Severe impairment	9 (5.2)	83.75 (18.75)			0.00 (0.00)		
Frailty status			35.176	0.000		39.255	0.000
Non-frail	13 (7.5)	36.25 (15.63)			175.00 (36.00)		
Prefrail	36 (20.8)	54.38 (30.63)			118.00 (70.50)		
Frail	124 (71.7)	66.25 (20.94)			39.50 (95.50)		
Depression			−10.362	0.000		−5.850	0.000
No	51 (29.5)	–			142.00 (97.00)		
Yes	122 (70.5)	–			40.00 (93.75)		

### 3.2. Comparison of depression scores with different demographic characteristics of participants

The median depression score in 173 older persons with OP was 62.5 (24.38). A total of 122 participants (70.5%) were depressed. There were significant differences in depression scores by age, cohabitation status, income, pain, comorbidity, polypharmacy, participation in social activities, ADL, and frailty status (*p* < 0.05) (Table 1).

### 3.3. Comparison of PA scores with different demographic characteristics of participants

The median PA score in 173 older persons with OP was 69.29 (116.64). There were statistically significant differences in PA scores according to age, marital status, education, cohabitation status, income, pain, polypharmacy, participation in social activities, ADLs, frailty status, and depression (*p* < 0.05) (Table 1).

### 3.4. Multivariate logistic regression analysis of factors related to depression

A multivariate logistic regression stepwise method was used to analyze the influencing factors of depression and showed that pain and PA were the main risk factors for depression in older persons with OP (*p* < 0.05). Participants with pain were 4.47 times more likely to be at risk of depression than those without pain. For each 1-point increase in PA score, the odds ratio for depression risk was 0.99 (Table 2).

**Table 2:** Multivariate logistic regression analysis of factors related to depression.

Variables	B	Sb	Wald	*P*	OR (95% CI)
Constant	−2.814	1.844	2.331	0.127	0.060
Pain	1.498	0.754	3.947	0.047	4.47 (1.02,19.61)
Polypharmacy	1.913	1.055	3.290	0.070	6.78 (0.86,53.58)
PA	−0.010	0.003	9.522	0.002	0.99 (0.98,1.00)
Frailty status					
Non-frail	−22.350	9770.451	0.000	0.998	0.00 (0.00)
Frail	−0.586	0.467	1.570	0.210	0.56 (0.22,1.39)

### 3.5. Multivariate linear regression analysis of factors related to PA

Multiple linear regression was used to analyze the effects of age, marital status, education, cohabitation status, income, smoking history, pain, history of fracture, polypharmacy, participation in social activities, ADLs, frailty status and depression on PA, and the final constructed multiple linear regression model had statistical significance (*F* = 2.397, *p* = 0.001). The model could explain 55.9% of the variance in PA (corrected *R*^2^ = 0.517). Female, participation in social activities, ADLs, and depression were the main risk factors for PA in older persons with OP (*p* < 0.05) (Table 3).

**Table 3:** Multivariate linear regression analysis of factors related to PA.

Independent variable	Unstandardized coefficients		Standardized regression coefficient	*t*	*P*
	Β	Standard error			
Constant	126.574	23.783		5.322	0.000
Female	24.148	10.169	0.128	2.375	0.019
Participation in social activities					
1–2 times/year	21.190	8.012	0.150	2.645	0.009
1–2 times/month	70.207	15.359	0.254	4.571	0.000
1–2 times/week	71.229	19.854	0.200	3.588	0.000
ADLs					
Mild impairment	−46.065	8.799	−0.328	−5.235	0.000
Moderate impairment	−99.170	14.083	−0.431	−7.042	0.000
Severe impairment	−90.805	18.469	−0.287	−4.917	0.000
Depression	−41.657	8.786	−0.270	−4.741	0.000

### 3.6. Analysis of the correlation between depression and PA

**Figure 1:**
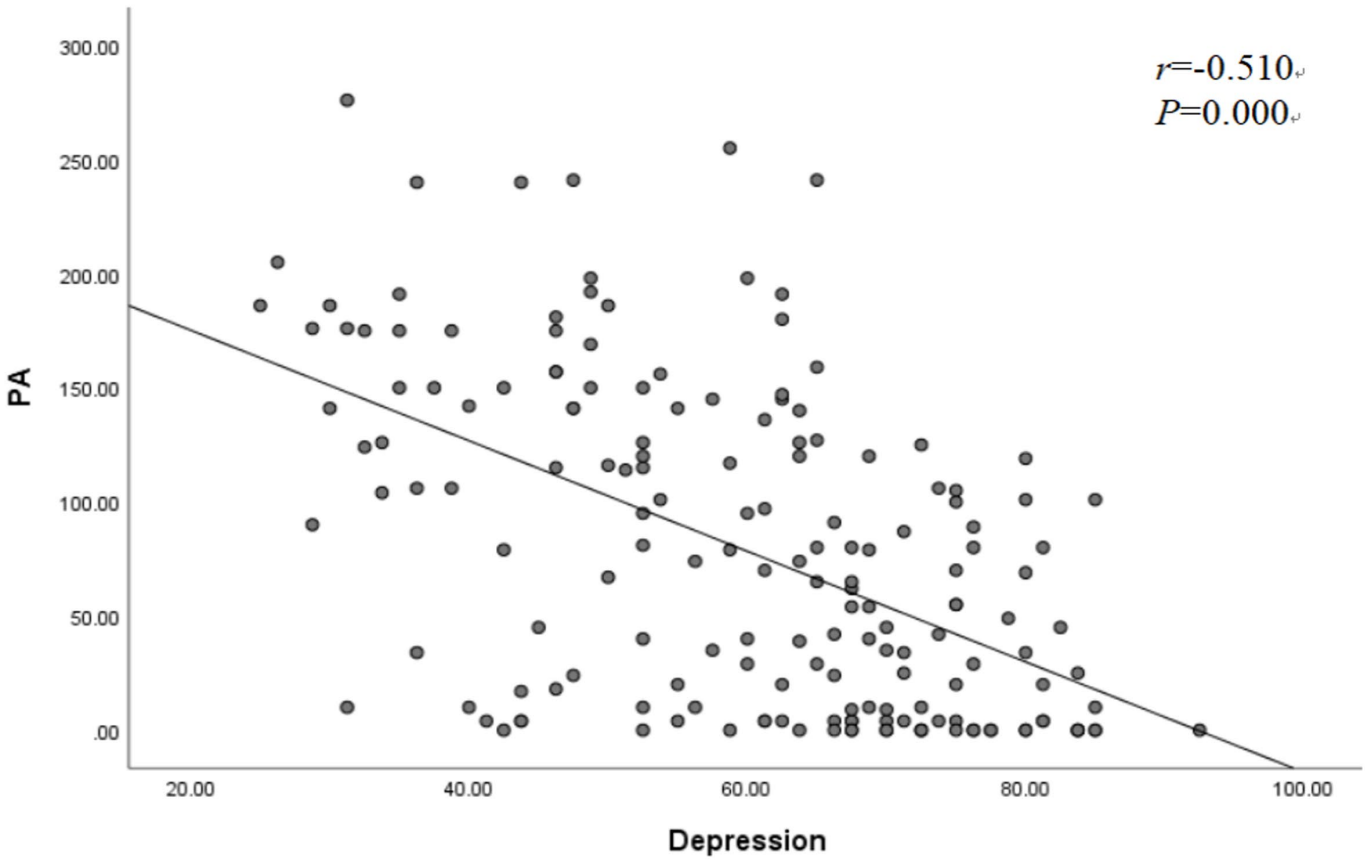
Correlation between PA and depression, *n* = 173.

Depression and PA scores showed a nonnormal distribution; therefore, Spearman correlation coefficients were calculated to analyzed the correlation between depression and PA. The results of the correlation analysis showed that depression was negatively correlated with PA (*R =* − 0.510*, p* = 0.000). This means that the higher the PA scores were, the lower the depression scores (Figure 1).

## 4. Discussion

The present study showed that depression was prevalent among a group of older persons with OP in China. This prevalence was higher than that reported in previous studies (14, 15, 34). The possible reason is that OP easily causes pain, weakness and fracture, resulting in limited activities and reduced social interaction. In addition, the subjects of this study were elderly individuals, who are more likely to have psychological problems. Our study showed that pain and PA were the main influencing factors related to depression in older persons with OP (Table 2). Persons with pain were 4.47 times more likely to be at risk of depression than those without pain, and this result was consistent with other study findings (35). Chronic pain could increase the risk for depression from 2.5 to 4.1 times in elderly individuals (36). Pain and depression are intertwined with each other, thus exacerbating physical and psychological symptoms. Pain reduces serotonin and norepinephrine. Decreases in these two neurotransmitters affect mood (37). Another study showed that the higher the pain scores were, the more serious the depression in older patients with OP (38). Pain can cause reduced mobility and limited daily activities, which may result in decreased interpersonal relationships, leading to depression (38). For each 1-point increase in PA score, the odds ratio for depression risk was 0.99. PA affects apoptosis and autophagy by releasing exercise-stimulated myohormone and the secretion of anti-inflammatory cytokines *via* mechanical force, helps to increase bone formation and relieve OP in the elderly (39). PA causes a wide range of biological changes in the brain and may exert some of its antidepressant effects by influencing cortisol responsiveness and hypothalamic–pituitary–adrenal (HPA) axis responses (40). Depression has been linked to dysregulation of the HPA axis (41). A systematic review reported that exercise can reduce depressive symptoms, which was indicated by moderate-to-large effect sizes, and it can be a useful complement to medication and psychotherapy (42). In addition, PA can improve self-esteem and life satisfaction, reduce loneliness, and promote psychosocial development and emotional balance in older adults (20).

We found that female, participation in social activities, ADLs, and depression were the main influencing factors related to PA in elderly patients with OP (Table 3). Housework involving PA, such as cleaning, cooking, gardening, and shopping, can benefit older people’s health (43). Females spend more time on housework than men, which may improve muscle strength and cognitive ability and increase PA scores (44). Social isolation was associated with depressive symptoms and psychological distress among older adults (45, 46). Our study showed that older persons who participated pin social activities 1–2 times/year, 1–2 times/month, and 1–2 times/week had higher PA scores than those who had no participation in social activities, which means that the more participation in social activities older persons have, the higher their PA scores. Previous studies have shown that social relationships can promote PA. Family and friends may encourage older persons to engage in more PA or may influence older persons through their own behavior, such as visiting and traveling with friends (45). PA is closely related to the physical health of elderly individuals, and impaired ADLs may limit PA (46). Older persons with OP have higher levels of comorbid depression, chronic somatic non-musculoskeletal diseases, and more pain than older adults without OP; these problems worsen the impairment of ADLs and result in decreased PA (18). ADLs are primarily dependent on motor functions, such as limb control and postural stability, which are necessary to accomplish the most basic forms of self-care. ADL injuries are often caused by musculoskeletal failure and render basic activities impossible, which results in a decrease in PA (47). Our study showed that participants with depression had lower PA, and these results were similar to those of other studies (35, 48). Depression can amplify pain and limit mobility (37). Older adults may reduce PA as a result of pain, fear of fractures, and increased depression, which may lead to decreased BMD and an increased risk of fractures (14).

Our results also showed that PA was negatively related to depression in older persons with OP (Figure 1). This means that lower PA levels may increase the risk of depression, and depression may decrease levels of activity due to low motivation and energy, thereby reducing PA; these results are similar to another study (38). In other words, depressed persons with OP are usually more prone to a sedentary lifestyle, consequently aggravating bone loss, and older persons with lower PA might be afraid to participate in their favorite forms of exercise, leading to a decrease in outdoor activities, which could negatively influence their mood and increase the risk of depression (49). A previous study showed that PA interventions can reduce depression and the impaired function caused by OP in older adults (50). The interaction between depression and PA may influence OP, and further research is still needed.

## 5. Limitations

Our study has some limitations that need to be considered. The main limitation of our study is the convenience sample, if we consider that we are referring to older persons with OP in China. Although all participants lived in southwestern China, they had different living conditions: some lived with children, while some did not, and some were married, while some were not. Because each region’s economy and way of life are different, our results may not be generalizable to other cultures or other geographic regions in China. The subjects in this investigation were older adults with OP, and the results of the depression and PA assessments may be different from those in older persons without OP. We should conduct multiregional and multicenter studies to improve the universality of the data samples in the future.

## 6. Conclusion

We found that depression was prevalent and that PA was negatively correlated with depression in older persons with OP in China. This means that the higher the PASE scores were, the lower the incidence of depression. Therefore, medical staff should give attention to depression, PA and its influencing factors; provide early screening and prompt identification of depression and PA among older persons with OP; and take effective measures to intervene and prevent adverse health outcomes. Further research on this topic is needed to gain deeper insight into these relationships.

## Data availability statement

The original contributions presented in the study are included in the article/supplementary material, further inquiries can be directed to the corresponding authors.

## Ethics statement

All methods were carried out in accordance with relevant guidelines and regulations regarding ethical approval and consent to participate. The ethics committee of the West China Hospital of Sichuan University approved this study and its methods in December 2020 [approval number 2020(59)]. All participants provided signed informed consent.

## Author contributions

RF carried out the project conceptualization, administration, and supervision. LS, XZ, XL, and YG performed the investigation and prepared the original draft. XD analyzed and interpreted the data. RF and LS reviewed and edited the manuscript. All authors read and approved the final manuscript.

## Funding

This study was supported by the West China Nursing Discipline Development Special Fund Project, Sichuan University, China (grant no. HXHL19021).

## Conflict of interest

The authors declare that the research was conducted in the absence of any commercial or financial relationships that could be construed as a potential conflict of interest.

## Publisher’s note

All claims expressed in this article are solely those of the authors and do not necessarily represent those of their affiliated organizations, or those of the publisher, the editors and the reviewers. Any product that may be evaluated in this article, or claim that may be made by its manufacturer, is not guaranteed or endorsed by the publisher.

## Abbreviations

OP: osteoporosis
PA: physical activity;
BMD: bone mineral density
DEXA: dual-energy X-ray absorptiometry;
SDS: Self-rating Depression Scale
PASE: Physical Activity Scale in the Elderly
ADLs: activities of daily living
