# Corrigendum: Evaluation of the correlation between depression and physical activity among older persons with osteoporosis: a cross-sectional study

**DOI:** 10.3389/fpsyt.2023.1340469

**Published:** 2024-01-15

**Authors:** Linjun Shi, Xiaoping Zhou, Yueshan Gao, Xia Li, Ronghua Fang, Xuexue Deng

**Affiliations:** ^1^West China School of Nursing/General Practice Ward, International Medical Center Ward, General Practice Medical Center, West China Hospital, Sichuan University, Chengdu, China; ^2^West China School of Nursing, Sichuan University, Chengdu, China; ^3^West China School of Nursing/Department of Nursing, West China Hospital, Sichuan University, Chengdu, China

**Keywords:** osteoporosis, older persons, depression, physical activity, cross-sectional study

In the published article, there were errors in the affiliations. Affiliation 3 was missing, and the incorrect affiliation numbers were applied to all authors. The correct affiliations appear above.

The authors apologize for these errors and state that this does not change the scientific conclusions of the article in any way. The original article has been updated.

